# Proton Pump Inhibitors and the Risk for Fracture at Specific Sites: Data Mining of the FDA Adverse Event Reporting System

**DOI:** 10.1038/s41598-017-05552-1

**Published:** 2017-07-17

**Authors:** Liwei Wang, Mei Li, Yuying Cao, Zhengqi Han, Xueju Wang, Elizabeth J. Atkinson, Hongfang Liu, Shreyasee Amin

**Affiliations:** 1 Department of Medical Informatics, School of Public Health, Jilin University, Changchun, 130021 Jilin Province China; 2 Department of Health Sciences Research, Mayo Clinic College of Medicine, Rochester, 55901 MN USA; 3 National Science Library, Chinese Academy of Science, Beijing, 100190 China; 4 Department of Pathology, The Third Hospital of Jilin University, Changchun, 130033 Jilin province China; 5 Division of Rheumatology, Mayo Clinic College of Medicine, Rochester, 55901 MN USA

## Abstract

Proton pump inhibitors (PPIs) are widely used to treat gastric acid-related disorders. Concerns have been raised about potential fracture risk, especially at the hip, spine and wrist. However, fracture risk at other bone sites has not been as well studied. We investigated the association between PPIs and specific fracture sites using an aggregated knowledge-enhanced database, the Food and Drug Administration Adverse Event Reporting System Data Mining Set (AERS-DM). Proportional reporting ratio (PRR) was used to detect statistically significant associations (signals) between PPIs and fractures. We analyzed both high level terms (HLT) and preferred terms (PT) for fracture sites, defined by MedDRA (Medical Dictionary for Regulatory Activities). Of PPI users reporting fractures, the mean age was 65.3 years and the female to male ratio was 3.4:1. Results revealed signals at multiple HLT and PT fracture sites, consistent for both sexes. These included fracture sites with predominant trabecular bone, not previously reported as being associated with PPIs, such as ‘rib fractures’, where signals were detected for overall PPIs as well as for each of 5 generic ingredients (insufficient data for dexlansoprazole). Based on data mining from AERS-DM, PPI use appears to be associated with an increased risk for fractures at multiple sites.

## Introduction

Proton pump inhibitors (PPIs) are acid suppressive agents used for managing gastric acid-related disorders, such as gastroesophageal reflux disease and peptic ulcers^1–3^. PPIs are among the most widely prescribed drugs; in the United States (US), PPIs were the third largest-selling therapeutic class and the 6th most widely dispensed retail prescription medications in 2008^4^.

The first PPI introduced, omeprazole, has been on the pharmaceutical market since 1989. Subsequently, lansoprazole, rabeprazole and pantoprazole successively entered into clinical practice^5^. In 2001, esomeprazole, a left-handed (S)-isomer of omeprazole, was introduced and then was widely used, ranking 4th in the top 20 drug list by sales in the global market in 2012^6, 7^. The newest PPI, dexlansoprazole, a right-handed (R)-isomer of lansoprazole, was approved in the US in 2009^7^.

In recent years, concerns have been raised about potential adverse drug events (ADEs) associated with chronic PPI use, including fractures, hypomagnesaemia, interstitial nephritis, iron and vitamin B12 malabsorption, and infections^8^. Among these ADEs, fractures have received increasing attention since 2006 when Vestergaard *et al*.^9^ and Yang *et al*.^10^ reported that PPI use was associated with an increased risk of fractures, specifically at the proximal femur (hip**)** ^9, 10^ and spine^9^. Since then, several studies have explored the association between PPIs and fracture risk^10–16^. The most recent meta-analysis has suggested that there is an increased risk of fractures at the hip, spine and overall fractures with PPI use^3^. However, results from some studies do not support an association between PPI and bone fragility^17^. In May 2010, the US Food and Drug Administration (FDA) issued the warning that PPI use had a possible increased risk of fractures at the hip, spine and distal forearm (wrist)^18^.

However, the risk of chronic PPI use for fractures at other specific sites has not been as well determined. Studies that examined the association between PPIs and fractures other than at the hip, spine, and wrist have reported only on overall fracture risk, i.e., overall (any) fracture^9^, any-site fractures^19–25^. One paper focusing on fractures in hepatitis patients using PPIs did list specific fracture sites, however, only the association with overall fracture risk was reported^26^.

Spontaneous reporting systems (SRSs), primarily used for postmarketing drug safety surveillance before epidemiological investigation, can provide additional information about ADEs, including fractures. Data mining algorithms have been developed for signal detection in SRSs for ADEs, where a ‘signal’ means a possible association between a drug and an ADE^27^. One of the popular signal detection methods is proportional reporting ratio (PRR)^28^, typically calculated to summarize the extent to which a particular adverse event is associated with a specific drug, compared to the background rate at which the same adverse event is associated with other drugs^29^. PRR is computed by building a 2×2 contingency table^28, 30^. The number of reports with the drug of interest and the event is defined as *a*. The number of reports with the drug of interest and without the event is assigned as *b*. The number of reports with drugs other than the drug of interest and with the event is defined as *c*. The number of reports with drugs other than the drug of interest and without the event is defined as *d*. A signal is detected when the reports of the drug associated with ADEs are 3 or more, and the PRR is at least 2 with the chi-squared of 4 or more^28^

1 $${\rm{PRR}}=\frac{a/(a+b)}{c/(c+d)}$$

AERS-DM (Adverse Event Reporting System Data Mining Set) is a normalized knowledge-enhanced data mining set^31^ of FDA’s Adverse Event Reporting System (FAERS), that is one of the commonly used databases to detect signals through data mining algorithms^32, 33^. Compared to FAERS, AERS-DM is more conducive to data mining with normalization and aggregation^34^. Based on this large-scale data set, we previously detected 668 drugs used in the 20 most frequent treatment regimens for the most common conditions in the US, and revealed sex differences in ADEs among 307 drugs corresponding to 736 statistically significant signals, some of which have been reported in drug labels and verified^35^. We also built a knowledge base of severe adverse events leveraging AERS-DM and semantic web technologies^36^. In another study, we have also demonstrated the feasibility to integrate AERS-DM with electronic health records (EHRs), and profiled ADEs of cancer drug ingredients using information from the Mayo Clinic EHRs^37^.

In this study, we aim to leverage the unique resources of AERS-DM to investigate the association between PPIs and ADEs reported at specific fracture sites using the data mining method PRR.

## Results

Among 2,459,001 reports in AERS-DM, 169,563 included at least one generic ingredient of PPIs, of which 3,782 are associated with fractures. Omeprazole, among PPIs, was the most frequently reported generic ingredient associated with all ADEs, including fractures, accounting for 33.7% and 34.0% of total reports and fracture reports, respectively, followed by esomeprazole (Table 1).

**Table 1:** Characteristics of the 169,563 MedDRA Adverse Event Reports involving at least one proton pump inhibitor (PPI) ingredient, comparing reports with a fracture and all reports with ADEs.

Characteristic	Reports with a fracture (%) N = 3,782	All reports (%) N = 169,563
Age (median), years		
0–19 (7)	14 (0.4%)	3,466 (2.0%)
20–29 (24)	24 (0.6%)	3,953 (2.3%)
30–39 (36)	87 (2.3%)	7,706 (4.5%)
40–49 (46)	271 (7.2%)	16,783 (9.9%)
50–59 (55)	627 (16.6%)	28,932 (17.1%)
60–69 (64)	809 (21.4%)	33,086 (19.5%)
70–79 (75)	768 (20.3%)	27,397 (16.2%)
80 + (84)	510 (13.5%)	15,427 (9.1%)
missing	672 (17.8%)	32,813 (19.4%)
Self-report	438 (11.58%)	15,273 (9.01%)
Females (%)	2,895 (76.5%)	100,192 (59.1%)
Self-report by female	361	10,652
Males (%)	851 (22.5%)	65432 (38.6%)
Self-report by male	77	4,553
Active PPI Ingredient*		
Omeprazole	1,421 (34.0%)	59,356 (33.7%)
Esomeprazole	1,220 (29.2%)	49,678 (28.2%)
Pantoprazole	748 (17.9%)	30,142 (17.1%)
Lansoprazole	607 (14.5%)	29,486 (16.7%)
Rabeprazole	180 (4.3%)	7,552 (4.3%)
Dexlansoprazole	9 (<0.1%)	59 (<0.1%)

*More than one generic ingredient could be reported in the reports.

**Figure 1:**
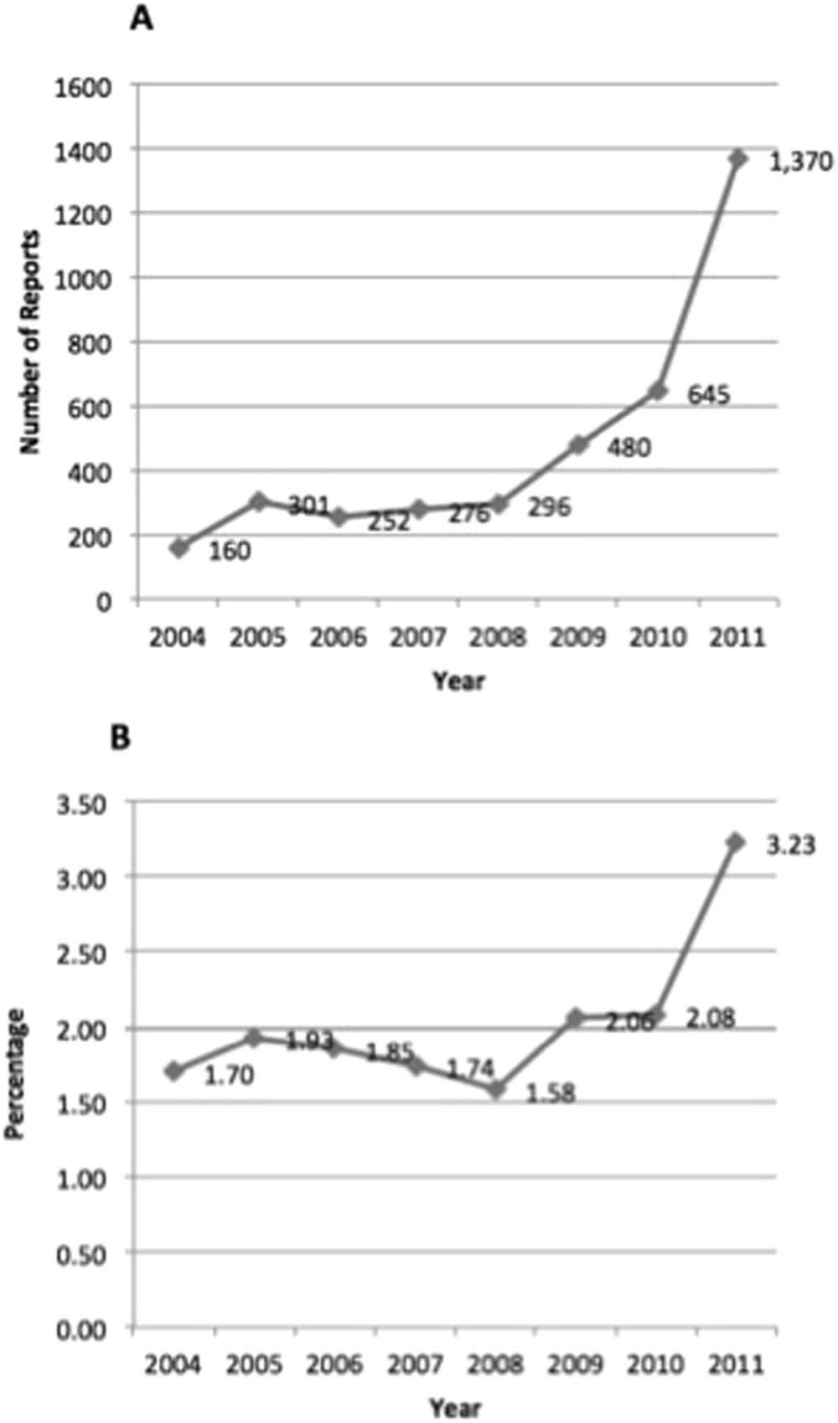
Increasing trend of proton pump inhibitor (PPI)-fracture reports from 2004 to 2011. (**A**) Reports of PPI with fractures. (**B**) Percentage of PPI-fracture reports among all PPI-AEs reports.

Information was available on sex in 3,742 (99%) PPI fracture reports and on age in 3,110 (82%). The mean age was 65.3 years (range: < 1 to ~99 years) and the ratio of females to males was 3.4:1. The age group of 60 to 69 years had the most ADE reports on fractures (21.4%) (Table 1). An increasing trend was observed for the reporting of fractures with PPI use and its percentage among all PPI-associated ADEs, especially after 2010 when the FDA black box warning was issued (Fig. 1).

At the *drug class-ADE class* level, there were signals detected for 4 of the 8 HLT fracture sites ‘thoracic cage fractures non-spinal’, ‘pelvic fractures’, ‘pathological fractures and complications’ and ‘spinal fractures’ (Table 2), of which the first three HLT categories of fracture sites have not previously been specifically reported. When analyses were stratified by age group, these signals were consistently observed in the 50–69 years and ≥ 70 years age groups but not in the age group ≤ 49 years.

**Table 2:** Signal detection between any proton pump inhibitor (PPI) and reported fracture adverse events as classified by MedDRA’s 8 High Level Terms (HLT) and corresponding 61 Preferred Terms (PT), by overall and age groups.

Fracture Site	Proportional Rate Ratio (PRR) by Age Groups (years)							
	Overall		≤49		50–69		≥70	
	N	PRR(χ^2^)	N	PRR(χ^2^)	N	PRR(χ^2^)	N	PRR(χ^2^)
Skull and face fractures	113	1.7(29.5)	22	1.2(0.6)	35	1.9(12.1)	39	**2.3(25.5)**
Skull fractured base	8	**2.8(7.9)**	2	2.1(1.0)	3	**5.1(7.1)**	3	**3.7(4.6)**
Skull fracture	24	1.2(0.7)	8	1.2(0.2)	5	0.9(<0.1)	7	1.5(1.1)
Facial bones fracture	81	1.8(25.7)	12	1.0(<0.1)	27	**2.0(12.2)**	29	**2.5(22.5)**
Fractured skull depressed	0	—	0	—	0	—	0	—
Thoracic cage fractures non-spinal (excl pathological)	437	**2.5(323.5)**	52	1.6(11.8)	165	**2.6(139.0)**	137	**2.9(143.0)**
Sternal fracture	22	1.9(9.5)	6	**2.8(5.7)**	11	**3.1(12.7)**	3	1.0(<0.1)
Rib fracture	423	**2.5(325.4)**	47	1.6(8.9)	160	**2.6(136.9)**	135	**3.0(151.3)**
Flail chest	2	2.5(1.5)	0	—	1	13.5(5.8)	1	2.3(0.6)
Spinal fractures (excl pathological)	517	**2.2(290.7)**	52	1.6 (9.3)	181	**2.6(155.4)**	188	**2.4(136.5)**
Spinal fracture	323	**2.3(209.9)**	30	1.6(5.4)	108	**2.9(112.0)**	117	**2.5(91.4)**
Cervical vertebral fracture	50	1.8(16.3)	7	1.3(0.5)	14	1.9(5.3)	17	**2.5(12.6)**
Thoracic vertebral fracture	58	**2.6(47.7)**	4	1.1(0.1)	26	**3.2(31.7)**	21	**2.9(21.0)**
Lumbar vertebral fracture	68	1.9(27.3)	7	1.7(1.9)	29	**2.6(23.5)**	24	1.9(8.7)
Fractured sacrum	43	**3.6(64.0)**	5	**3.1(5.7)**	15	**3.4(20.1)**	18	**5.0(41.6)**
Fractured coccyx	13	1.2(0.6)	3	1.2(0.1)	4	1.1(<0.1)	3	1.1(<0.1)
Pelvic fractures	200	**2.1(98.4)**	11	1.0(<0.1)	54	**2.2(30.3)**	90	**2.3(55.1)**
Pelvic fracture	160	1.9(62.6)	10	1.2(0.2)	40	**2.0(18.0)**	70	1.9(29.0)
Ilium fracture	4	1.4(0.3)	0	—	2	1.8(0.6)	1	1.4(0.1)
Pubis fracture	26	**3.8(41.5)**	0	—	11	**4.4(21.6)**	13	**4.2(24.0)**
Acetabulum fracture	14	**3.6(21.1)**	1	0.9(0.01)	4	2.8(3.9)	7	**7.9(26.6)**
Fractured ischium	2	1.0(<0.1)	0	—	1	1.2(<0.1)	1	1.5(0.2)
Upper limb fractures	768	1.8(249.3)	87	1.2(3.4)	287	1.9(123.5)	270	**2.4(179.5)**
Upper Limb fracture	282	1.7(69.6)	22	0.9(0.3)	106	1.8(34.2)	108	**2.5(81.9)**
Clavicle fracture	57	1.7(15.1)	11	1.3(0.9)	20	1.9(8.2)	15	**2.1(7.8)**
Scapula fracture	14	**2.7 (12.5)**	3	2.5(2.3)	8	**5.4(20.5)**	2	1.6(0.4)
Humerus fracture	114	**2.2(61.4)**	10	1.3(0.6)	44	**2.5(32.7)**	41	**2.3(25.7)**
Forearm fracture	10	1.8(3.3)	2	2.1(1.0)	6	**4.3(11.4)**	2	1.1(<0.1)
Radius fracture	65	**2.6(51.6)**	10	1.9(3.9)	23	**2.4(15.8)**	26	**3.6(38.3)**
Ulna fracture	30	**3.0(32.3)**	5	2.3(3.2)	17	**5.3(43.0)**	4	1.3(0.3)
Wrist fracture	205	1.9(78.7)	21	1.5(3.2)	75	1.9(28.4)	82	**2.6(66.4)**
Hand fracture	100	1.9(38.9)	24	1.8(7.3)	34	1.9(11.7)	20	**2.3(12.9)**
Scapulothoracic dissociation	0	—	0	—	0	—	0	—
Lower limb fractures	1714	1.5(270.0)	161	1.1(1.6)	662	1.7(177.0)	609	1.9(237.7)
Lower limb fracture	202	1.4(24.0)	25	0.9(0.1)	94	1.8(30.7)	41	1.6(7.8)
Hip fracture	497	1.7(136.7)	23	1.4(2.4)	121	1.6(24.4)	264	**2.3(159.9)**
Femoral neck fracture	81	1.5(12.5)	4	1.0(0.0)	25	**2.1(13.1)**	43	1.5(5.8)
Femur fracture	454	1.3(24.8)	32	1.4(2.9)	203	1.6(40.2)	177	1.7(44.1)
Patella fracture	35	1.7(8.2)	3	0.9(0.1)	17	**2.1(8.3)**	10	1.9(3.4)
Tibia fracture	92	1.9(37.8)	9	0.8(0.6)	42	**2.1(21.8)**	29	**3.6(42.3)**
Fibula fracture	71	**2.2(39.7)**	11	1.4(0.9)	34	**2.4(23.1)**	14	**2.5(10.8)**
Ankle fracture	223	1.5(31.1)	22	0.6(4.7)	103	1.6(23.3)	42	1.8(12.4)
Foot fracture	291	1.8(97.1)	56	1.5(8.4)	149	**2.2(79.3)**	39	1.9(15.8)
Fractures NEC (excl pathological)	638	1.9(277.9)	80	1.7(18.8)	269	**2.6(225.7)**	185	**2.3(115.8)**
Periprosthetic fracture	4	**4.9(9.1)**	0	—	2	3.9(3.3)	2	13.5(11.6)
Fracture displacement	18	**4.2(33.4)**	1	2.7(0.9)	8	**3.6(11.9)**	8	**5.4(20.5)**
Compression fracture	169	**3.7(256.9)**	17	**4.2(31.4)**	73	**5.4(185.3)**	53	**2.9(54.3)**
Bone fragmentation	28	**3.6(41.5)**	2	1.7(0.5)	18	**5.5(47.3)**	4	1.9(1.4)
Avulsion fracture	5	**2.7(4.5)**	1	1.4(0.1)	2	3.9(3.3)	2	4.5(4.1)
Jaw fracture	77	**2.5(61.0)**	12	1.7(3.2)	37	**2.9(38.5)**	17	**3.1(20.2)**
Bone fissure	6	1.3(0.3)	1	2.3(0.6)	1	0.4(0.7)	3	2.5(2.3)
Complicated fracture	3	3.5(0.1)	0	—	2	13.5(11.6)	1	3.4(1.3)
Epiphyseal fracture	1	3.4(1.3)	1	3.4(1.3)	0	—	0	—
Fracture	168	1.3(13.9)	12	1.0(0.0)	64	**2.3(38.1)**	51	1.6(11.4)
Impacted fracture	1	2.7(0.9)	0	—	0	—	0	—
Multiple fractures	59	1.7(15.0)	8	1.0(0.0)	23	**2.2(13.0)**	18	**2.5(14.1)**
Open fracture	13	1.9(5.1)	3	1.6(0.5)	3	1.3(0.2)	3	2.5(2.3)
Stress fracture	116	1.9(40.8)	17	**3.1(19.7)**	55	**2.0(24.0)**	30	**2.5(23.7)**
Torus fracture	1	3.4(1.3)	1	4.5(2.0)	0	—	0	—
Traumatic fracture	11	1.5(1.9)	0	—	4	1.5(0.6)	4	2.2(2.2)
Greenstick fracture	0	—	0	—	0	—	0	—
Pathological fractures and complications	277	**2.6(223.3)**	32	1.8(10.3)	119	**2.7(103.8)**	94	**3.6(137.4)**
Pathological fracture	188	**3.1(218.6)**	20	**2.5(14.9)**	78	**3.2(93.5)**	72	**4.3(137.6)**
Pseudarthrosis	8	**2.3(5.3)**	3	**4.1(5.3)**	4	**3.2(4.8)**	1	1.1(<0.1)
Osteoporotic fracture	31	**2.3(19.7)**	5	1.2(0.1)	7	**2.3(4.4)**	12	**3.7(18.4)**
Fracture nonunion	39	**2.2(21.8)**	4	1.6(0.9)	24	**2.3(15.8)**	6	2.2(3.3)
Fracture malunion	2	0.8(0.1)	0	—	1	0.8(<0.1)	0	—
Fracture delayed union	20	1.7(5.1)	1	0.9(0.01)	11	1.7(3.1)	7	**2.3(4.2)**
Synostosis	1	0.5(0.6)	0	—	0	—	1	—

Around 82% of reports have age information. Bold indicates statistically significant signals.

Analyses at the *drug class-ADEs* level yielded signals at 22 of the 61 PT fracture sites, which were represented under all 8 HLTs (Table 2). *Drug class-ADEs* signals for the PT ‘rib fracture’ primarily contributed to the *drug class-ADE class* signal of the HLT ‘thoracic cage fractures non-spinal’. While the signal for the *drug class-ADE class* HLT ‘upper limb fractures’ did not reach statistical significance (PRR = 1.8), several PT fracture sites under this category either did show a signal or had a PRR that approached a statistically significant signal, including for the PT ‘wrist fracture’ (PRR = 1.9). Furthermore, a signal was more likely to be observed at more PT sites within the HLT ‘upper limb fractures’, including for ‘wrist fracture’ and ‘humerus fracture’ when considering the two older age groups (Table 2). Similarly, within the HLT ‘lower limb fractures’, signals were more likely at PT sites in the two older age groups. Specifically, a signal was observed for ‘hip fracture’ in the ≥ 70 years group (PRR = 2.3) and for ‘femoral neck fracture’ (PRR = 2.1) in the 50–69 years group. Lastly, within the HLT ‘fractures NEC’ there was a signal in all age groups for the PT ‘compression fracture’, while there was also a trend for signals in the PT ‘stress fracture’. These two PTs were the most common fracture sites within this HLT.

Supplementary Table S1 provides additional details on signals detected in females and males, separately, for the 8 HLTs and corresponding 61 PTs. The majority of signals observed for the PT fracture sites tended to be consistent between females and males.

For the *drugs-ADEs* level, dexlansoprazole showed no signals (results not shown) most likely due to the late launch into the market relative to the time period covered in our analyses with fewer corresponding data available, as noted in Table 1. For the remaining five PPIs, there were a total 112 signals detected corresponding to 42 PT sites of fractures. Among these PT fracture sites, ‘rib fracture’, ‘pathological fracture’ and ‘compression fracture’ each showed signals for all five PPIs (Supplementary Table S2). There was also a trend for signals at the overall HLTs ‘upper limb fractures’ and ‘spinal fractures’, as well as the PTs ‘hip fracture’ and ‘stress fracture’ for all five PPIs.

## Discussion

To the best of our knowledge, this is the first study to explore associations between PPI use and fracture risk at all specific bone sites through data mining in a large spontaneous reporting database. Although statistically significant signals between PPI and fractures at multiple specific sites were detected in this study, it should be noted that the number of fracture reports was a small proportion (~2–3%) of the ADEs reported with PPIs.

We identified signals between the drug class PPI and three HLT fracture categories, as classified by MedDRA, ‘thoracic cage fractures non-spinal’, ‘pelvic fractures’ and ‘pathological fractures and complications’. Within the HLT ‘thoracic cage fractures non-spinal’, rib fractures were the most relevant. Our findings of a signal with the HLT ‘spinal fractures’ are also consistent with prior reports of an increased risk for spine fractures with PPI use. Similarly, when analyses were stratified by age groups, we identified signals in the older age groups between PPI use and several more specific PT fracture sites, including at the hip, wrist and humerus, typical sites for fragility fractures in this age range. Overall, the signals detected between the use of PPIs and risk for fracture at different sites appeared to be primarily driven by a greater risk in those over age 50 years.

A signal for the PT ‘pathological fractures’ was detected at all levels in this study. MedDRA has no clear definition for ‘pathological fractures’. In other studies, a pathologic fracture is a fracture caused by disease leading to weakness of the bone^38, 39^. The different processes leading to a pathological fracture may include osteitis, osteogenesis imperfecta, osteomalacia/rickets, Paget’s disease or primary bone tumors/ metastases, although ‘osteoporosis’ has sometimes been included in this list^39^. Indeed, the PT ‘osteoporotic fracture’ was included in the HLT ‘pathological fractures and complications’. It is not clear what exactly qualified as a pathologic fracture in these reports, but if PPIs have an adverse effect on bone metabolism, it may be exacerbating the risk of a fracture in bones already weakened by a localized malignancy.

The mechanism through which PPIs may increase the risk for fractures remains unclear. Studies have explored the potential effects of PPIs on calcium absorption^40^, bone mineral density (BMD)^41–44^, bone metabolism^45, 46^, increased histamine release^47^, or their association with fall risk^16, 23^. In addition, it has been hypothesized that PPI use may be associated with hyperparathyroidism^48^, which has been recently reported in a cohort of elderly subjects^49^. We further investigated the association between PPIs and hyperparathyroidism in AERS-DM, and did find a signal (PRR 3.1837, χ^2^ 40.001). It is possible that there may be multiple factors contributing to the association observed between PPIs and increased fractures. That the signals detected in our study seemed to be strongest in bones that were primarily trabecular (e.g., ribs, spine) may be particularly relevant in understanding the apparent adverse effect of PPIs on bone health. Indeed, Maggio *et al*., did suggest that PPIs may have an adverse effect on trabecular bone in particular^50^_._ We found fracture risk with PPI use was more likely in ages over 50 years. If PPI use is adversely affecting bone metabolism, an increase in fractures may not be detectable in younger ages, where bone strength may still be sufficient enough to protect against fracture from low-to-moderate trauma. Duration of PPI use was not available for our analyses.

With the increase in age of those reporting an ADE, the ratio between female and male reporting fractures also increased. A few existing studies have focused on sex differences between PPI use and risk for fractures, and found that the association was stronger in men^10, 14^. Other studies have focused on postmenopausal women^12, 20, 24, 51–53^, children or young adults^54^. It was found women are also more likely to have fractures than men with increasing age^55^. Here, we also looked into the self-report and gender distribution in FAERS for PPIs (Table 1). Overall, 15,273 out of all PPI reports, i.e., 9.01%, are self-reported with 438 (0.26%) self-reported fracture cases. 361 out of 438 fracture cases are females. The ratio of females to males is 4.69 for self-reported fracture cases. Among all PPI reports, 100,192 (59.09%) are females with 2,895 (1.71%) female fracture cases. The ratio of females to males is 3.26 for all PPI self-reports. Comparing with the ratio of females to males in FAERS, i.e., 1.58, our results showed more fracture cases in females than males. Nonetheless, the signals we identified at different fracture sites were mostly consistent between females and males. Confounding factors could still exist and further studies are warranted to determine if there are any sex differences between PPI use and risk for fractures.

For all studied fracture sites, we used terms from MedDRA. We found MedDRA still has areas to improve due to its lack of clear definitions for terms. For example, it would be more consistent with clinical practice if the PT term ‘compression fracture’ is included in the HLT ‘spinal fractures’ instead of ‘fractures NEC’, and the PT term ‘jaw fracture’ was included under the HLT ‘skull and face fractures’ instead of ‘fractures NEC’. Furthermore, we note that the PT ‘osteoporotic fracture’ was included under the HLT ‘pathologic fractures and complications’.

In this study, we used AERS-DM as the data source. The features of drug classification and ADE aggregation make it possible to detect signals at different levels^35^. However, the mined ADE signals do not indicate a causal relationship with PPIs and the incidence cannot be obtained from the spontaneous reporting system due to lacking the number of people exposed to PPIs. In addition, drug-drug interaction cannot be considered. Bias in reporting of ADEs, may also influence results. We did note an increase in reporting of fracture ADE with PPIs following the FDA black box warning in 2010. Confounding by concurrent comorbidities cannot be excluded when using AERS-DM. Therefore, data from randomized controlled trial studies or from other epidemiologic studies are needed to clarify the relationships observed when using AERS-DM. Nevertheless, data mining through such unique resources can provide valuable information on potential ADEs, including fractures, which would then require further investigation.

## Conclusions

The current study reveals associations between PPI use and multiple different sites of fractures based on data mining from AERS-DM. Our findings revealed an increased fracture risk at three previously unreported categories of fracture sites including ‘thoracic cage fractures non-spinal’, ‘pelvic fractures’ and ‘pathological fractures and complications’, in addition to the more recognized site of ‘spinal fractures’. Furthermore, signals were identified for multiple different fracture sites beyond these broader categories, including at the hip and wrist, with the PRR for many other sites approaching a statistically significant signal. These findings were primarily observed in individuals who were older, at least over the age of 50 years. Signals identified tended to also be consistent between women and men. These results suggest that PPI use may contribute to an adverse effect on bone metabolism, which may particularly influence trabecular bone. Further work, however, is necessary to better understand how PPI may increase fracture risk. Our findings would generate hypotheses for future experimental studies to investigate potential association between PPIs and risk for fracture at multiple sites, as well as underlying mechanism. Our work also illustrates the advantages of data mining the AERS-DM to help identify drugs that may potentially be associated with fractures.

## Methods

### Data sources

As a database supporting the post-marketing safety surveillance for drug and therapeutic biologic products^56^, FAERS distributes seven datasets regarding the information of each report, including DEMO (demographic and administrative information), DRUG (drug information), REAC (reaction information), OUTC (patient outcome information), RPSR (information on the source of the reports), THER (drug therapy start dates and end dates), and INDI (“Medical Dictionary for Regulatory Activities” [MedDRA] terms coded for the indications for use [diagnoses] for the reported drugs)^57^.

In FAERS, drugs can be registered by arbitrary names, including trade names, abbreviations, and even with typographical errors, further complicating downstream analysis. Previously, we standardized FAERS data through three steps: de-duplication, drug normalization, and data aggregation^31^. First, redundant reports were removed as suggested by the FDA. Second, FAERS drug names, along with administration route and dose information, were normalized using a natural language processing (NLP) tool MedEx^58^ to RxNorm, a standardized nomenclature for clinical drugs and drug delivery devices^59^. Meanwhile, adverse event terms were mapped to MedDRA’s preferred term (PT) code and classified into MedDRA System Organ Class (SOC)^60^. Third, adverse events were then aggregated according to MedDRA SOC and PT codes, and drugs aggregated based on National Drug File–Reference Terminology (NDF-RT) classification information through RxNorm^61^.

We processed FAERS data from 2004 through 2011 into AERS-DM, which contains 2,459,001 reports and 37,029,228 Drug-ADE records. In total, 74% of FAERS unique drug names were normalized to 14,489 unique RxNorm concepts, of which 10,221 (71%) were classified in NDF-RT. The datasets of AERS-DM can be downloaded from the website http://informatics.mayo.edu/adepedia/index.php/Download.

### Data extraction

MedDRA has a 5-level hierarchical structure, including System Organ Class (SOC), High Level Group Term (HLGT), High Level Term (HLT), Preferred Term (PT), and Low Level Term (LLT). Each high-level term list includes all sub-level terms. In order to extract PTs in AERS-DM, version 14.1 of MedDRA was utilized to extract the HLGT ‘Fractures’ with the code ‘10017322’ first. Then, 8 HLTs and 61 PTs and corresponding codes associated with fractures were extracted. The 8 HLTs were: 1) ‘skull and face fractures’, 2) ‘thoracic cage fractures non-spinal (excl pathological)’, 3) ‘spinal fractures (excl pathological)’, 4) ‘pelvic fractures’, 5) ‘upper limb fractures’, 6) ‘lower limb fractures’, 7) ‘fractures NEC [not elsewhere classified] (excl pathological)’ and 8) ‘pathological fractures and complications’. These 8 HLTs and their corresponding 61 PTs were used to report our results that are presented in Table 2 and in additional Supplementary Tables S1 and S2.

NDF-RT code ‘N0000000147’ for PPIs class as one of Cellular or Molecular Interactions was mapped to RxNorm code ‘986356’, that was then used to extract 33 associated RxNorm codes for sub-class drugs, including 6 PPI generic ingredients (omeprazole, esomeprazole, pantoprazole, lansoprazole, rabeprazole and dexlansoprazole).

Then the 61 fracture PTs codes and 6 PPI RxNorm codes were used to extract records from AERS-DM.

### Data mining

The data mining method PRR was used to detect associations between PPIs and specific fractures reported using the 61 PTs for fractures as well as the 8 HLTs. To build the contingency table for PRR, the number of reports with PPI and fracture is defined as *a*. The number of reports with PPI and without fracture is assigned as *b*. The number of reports with drugs other than PPI and with fracture is defined as *c*. The number of reports with drugs other than PPI and without fracture is defined as *d*. PRR was then calculated base on Formula 1.

Studies have shown that higher levels of MedDRA could strengthen the sensitivity of signal detection for revealing associations in groups^62, 63^. In this study, we evaluated the PRR for different levels to gain insight into associations between PPIs and specific fracture sites, including ***drug class-ADE class*** (between all PPIs and 8 fracture HLTs), ***drug class-ADEs*** (between all PPIs and 61 fracture PTs) and ***drugs-ADEs*** (between each PPI and 61 fracture PTs). As fracture risk is different between women and men, and with increasing age, we also stratified analyses for the *drug class-ADE class* and *drug class-ADEs*, by sex and by age groups ( ≤ 49 years, 50–69 years and ≥ 70 years).

## Electronic supplementary material

Supplementary Information
