# Supplementary material for: Proton Pump Inhibitors and the Risk for Fracture at Specific Sites: Data Mining of the FDA Adverse Event Reporting System
Source: Sci Rep. 2017 Jul 17;7:5527. doi: 10.1038/s41598-017-05552-1 (PMC5514095; doi:10.1038/s41598-017-05552-1)
Supplement: Supplementary file 1 — Supplementary Information [file 41598_2017_5552_MOESM1_ESM.pdf]

Proton Pump Inhibitors and the Risk for Fracture at Specific Sites: Data Mining of the FDA Adverse Event Reporting System

Liwei Wang[1,2,*], Mei Li[1], Yuying Cao[1], Zhengqi Han[3], Xueju Wang[4], Elizabeth J. Atkinson[2], Hongfang Liu[2,*], Shreyasee Amin[2, 5,*]

1 Department of Medical Informatics, School of Public Health, Jilin University, Changchun 130021, Jilin Province, China.
2 Department of Health Sciences Research, Mayo Clinic College of Medicine, Rochester 55901 MN, U.S.
3 National Science Library, Chinese Academy of Science, Beijing 100190, China
4 Department of Pathology, the Third Hospital of Jilin University, Changchun130033, Jilin province, China
5 Division of Rheumatology, Mayo Clinic College of Medicine, Rochester 55901 MN, U.S

Liwei Wang and Mei Li are co-first authors.
*Corresponding.author: wang.liwei@mayo.edu
*Corresponding author: liu.hongfang@mayo.edu
*Corresponding author: amin.shreyasee@mayo.edu

Page 2-4 Supplementary Table S1. Signal detection between any proton pump inhibitor (PPI) and reported fracture adverse events as classified by MedDRA's 8 High Level Terms (HLT) and corresponding 61 Preferred Terms (PT), by overall and gender groups. Bold indicates statistically significant signals.

Page 5-7 Supplementary Table S2. Signal detection between each proton pump inhibitor (PPI) and reported fracture adverse events as classified by MedDRA's 8 High Level Terms (HLT) and corresponding 61 Preferred Terms (PT). Bold indicates a statistically significant signal.

Supplementary Table S1. Signal detection between any proton pump inhibitor (PPI) and reported fracture adverse events as classified by MedDRA's 8 High Level Terms (HLT) and corresponding 61 Preferred Terms (PT), by overall and gender groups. Bold indicates statistically significant signals.

Fracture Site	Overall		Males		Females	
	N	PRR(χ^2)	N	PRR(χ^2)	N	PRR(χ^2)
Skull and face fractures	113	1.7(29.5)	32	1.2(0.9)	81	**2.2(44.1)**
Skull fractured base	8	**2.8(7.9)**	4	2.3(2.4)	4	**4.2(7.3)**
Skull fracture	24	1.2(0.7)	11	1.2(0.3)	13	1.2(0.5)
Facial bones fracture	81	1.8(25.7)	17	1.0(<0.1)	64	**2.4(45.6)**
Fractured skull depressed	0	—	0	—	0	—
Thoracic cage fractures non-spinal (excl pathological)	437	**2.5(323.5)**	143	**2.5(104.9)**	291	**2.6(231.5)**
Sternal fracture	22	1.9(9.5)	8	**2.6(6.5)**	14	1.9(5.3)
Rib fracture	423	**2.5(325.4)**	138	**2.5(103.2)**	282	**2.6(234.0)**
Flail chest	2	2.5(1.5)	1	2.7(0.9)	1	2.3(0.6)
Spinal fractures (excl pathological)	517	**2.2(290.7)**	114	**2.1(53.5)**	396	**2.3(244.9)**
Spinal fracture	323	**2.3(209.9)**	74	**2.4(53.7)**	245	**2.4(163.6)**
Cervical vertebral fracture	50	1.8(16.3)	16	1.5(2.1)	34	**2.1(16.9)**
Thoracic vertebral fracture	58	**2.6(47.7)**	7	**1.3(0.5)**	49	**3.0(52.0)**
Lumbar vertebral fracture	68	1.9(27.3)	13	1.4(1.2)	53	**2.2(28.6)**
Fractured sacrum	43	**3.6(64.0)**	7	**6.3(21.3)**	36	**3.4(47.6)**
Fractured coccyx	13	1.2(0.6)	3	2.0(1.4)	10	1.1(0.2)
Pelvic fractures	200	**2.1(98.4)**	31	**2.3(18.5)**	167	**2.1(84.2)**
Pelvic fracture	160	1.9(62.6)	22	**2.0(9.7)**	136	1.9(55.5)
Ilium fracture	4	1.4(0.3)	2	3.9(3.3)	2	0.9(<0.1)
Pubis fracture	26	**3.8(41.5)**	4	**6.0(11.5)**	22	**3.5(31.7)**

Acetabulum fracture	14	**3.6(21.1)**	3	1.8(1.0)	11	**4.9(25.4)**
Fractured ischium	2	1.0(<0.1)	0	—	2	1.5(0.3)
Upper limb fractures	768	1.8 (249.3)	149	1.7(35.4)	615	1.9(232.4)
Upper Limb fracture	282	1.7(69.6)	48	1.5(6.6)	234	1.8(72.5)
Clavicle fracture	57	1.7 (15.1)	19	1.7(4.6)	37	1.8(11.4)
Scapula fracture	14	**2.7 (12.5)**	9	**5.1(21.4)**	5	1.5(0.8)
Humerus fracture	114	**2.2(61.4)**	17	1.5(2.4)	97	**2.4(67.2)**
Forearm fracture	10	1.8(3.3)	1	0.9(<0.1)	9	**2.2(5.1)**
Radius fracture	65	**2.6(51.6)**	16	**2.8(15.8)**	49	**2.5(37.4)**
Ulna fracture	30	**3.0(32.3)**	8	**4.3(15.5)**	22	**2.7(19.6)**
Wrist fracture	205	1.9(78.7)	26	1.9(9.2)	177	1.9(72.1)
Hand fracture	100	1.9(38.9)	22	1.4(2.6)	77	**2.2(42.6)**
Scapulothoracic dissociation	0	—	0	—	0	—
Lower limb fractures	1714	1.5(270.0)	320	1.6(61.2)	1376	1.5(231.9)
Lower limb fracture	202	1.4(24.0)	40	1.1(0.5)	161	1.6(30.3)
Hip fracture	497	1.7(136.7)	92	1.6(18.7)	402	1.8(130.2)
Femoral neck fracture	81	1.5(12.5)	17	1.2(0.7)	63	1.6(14.2)
Femur fracture	454	1.3(24.8)	61	1.7(14.6)	390	1.3(21.4)
Patella fracture	35	1.7(8.2)	5	1.5(0.7)	30	1.7(8.5)
Tibia fracture	92	1.9(37.8)	19	1.8(6.5)	71	**2.0(31.2)**
Fibula fracture	71	**2.2(39.7)**	15	1.9(6.4)	54	**2.2(31.9)**
Ankle fracture	223	1.5(31.1)	36	1.3(1.9)	181	1.5(27.7)
Foot fracture	291	1.8(97.1)	66	**2.5(52.2)**	224	1.7(62.5)
Fractures NEC (excl pathological)	638	1.9(277.9)	149	**2.0(67.6)**	484	**2.1(233.1)**
Periprosthetic fracture	4	**4.9(9.1)**	0	—	4	**5.4(10.3)**
Fracture displacement	18	**4.2(33.4)**	0	—	18	**4.7(38.6)**

Compression fracture	169	**3.7(256.9)**	45	**3.9(75.9)**	124	**3.7(189.7)**
Bone fragmentation	28	**3.6(41.5)**	7	**3.2(8.3)**	21	**4.1(37.1)**
Avulsion fracture	5	**2.7(4.5)**	3	**3.4(4.0)**	2	2.3(1.2)
Jaw fracture	77	**2.5(61.0)**	27	**2.8(25.2)**	50	**2.5(38.9)**
Bone fissure	6	1.3(0.3)	2	1.9(0.8)	4	1.1(0.1)
Complicated fracture	3	3.5(0.1)	0	—	3	**6.8(9.8)**
Epiphyseal fracture	1	3.4(1.3)	1	6.8(3.3)	0	—
Fracture	168	1.3(13.9)	32	1.1(0.3)	134	1.5(21.4)
Impacted fracture	1	2.7(0.9)	1	6.8(3.3)	0	—
Multiple fractures	59	1.7(15.0)	19	1.8(5.8)	40	1.7(10.5)
Open fracture	13	1.9(5.1)	6	**2.8(5.7)**	7	1.6(1.4)
Stress fracture	116	1.9(40.8)	19	**3.7(29.6)**	95	1.7(24.3)
Torus fracture	1	3.4(1.3)	0	—	1	6.8(3.3)
Traumatic fracture	11	1.5(1.9)	1	0.6(0.2)	9	1.7(2.5)
Greenstick fracture	0	—	0	—	0	—
Pathological fractures and complications	277	**2.6(223.3)**	75	**3.2(92.0)**	201	**2.5(147.6)**
Pathological fracture	188	**3.1(218.6)**	55	**3.6(83.0)**	133	**3.0(147.7)**
Pseudarthrosis	8	**2.3(5.3)**	4	**3.6(5.9)**	4	1.9(1.6)
Osteoporotic fracture	31	**2.3(19.7)**	8	**2.5(6.1)**	23	**2.3(15.0)**
Fracture nonunion	39	**2.2(21.8)**	6	**3.7(9.2)**	32	**2.0(14.1)**
Fracture malunion	2	0.8(0.1)	1	13.5(5.8)	1	0.5(0.6)
Fracture delayed union	20	1.7(5.1)	2	2.3(1.2)	18	1.7(4.6)
Synostosis	1	0.5(0.6)	0	—	1	1.5(0.2)

Supplementary Table S2. Signal detection between each proton pump inhibitor (PPI) and reported fracture adverse events as classified by MedDRA's 8 High Level Terms (HLT) and corresponding 61 Preferred Terms (PT). Bold indicates a statistically significant signal.

	Omeprazole		Lansoprazole		Pantoprazole		Rabeprazole		Esomeprazole	
	Reports	PRR(χ^2)	Reports	PRR(χ^2)	Reports	PRR(χ^2)	Reports	PRR(χ^2)	Reports	PRR(χ^2)
Skull and face fractures	47	1.9(21.8)	17	1.4(2.0)	**25**	**2.1(13.2)**	**14**	**4.6(38.7)**	29	1.4(3.8)
Skull fractured base	**4**	**3.9(7.7)**	0	—	0	—	0	—	**4**	**4.6(10.4)**
Skull fracture	11	1.6(2.1)	3	0.8(0.1)	3	0.8(0.1)	1	1.1(<0.1)	8	1.3(0.7)
Facial bones fracture	32	1.9(14.7)	14	1.7(4.1)	**22**	**2.7(22.2)**	**13**	**6.3(56.4)**	17	1.2(0.7)
Fractured skull depressed	0	—	0	—	0	—	0	—	0	—
Thoracic cage fractures non-spinal (excl pathological)	**181**	**2.1(122.8)**	**75**	**2.2(50.5)**	**101**	**3.0(128.8)**	**19**	**2.2(12.3)**	**81**	**2.4(61.7)**
Sternal fracture	**10**	**2.5(8.6)**	2	1.0(<0.1)	**5**	**2.4(4.1)**	0	—	6	1.8(1.9)
Rib fracture	**175**	**2.8(190.5)**	**73**	**2.3(51.8)**	**98**	**3.0(129.4)**	**19**	**2.3(13.9)**	**135**	**2.6(121.7)**
Flail chest	0	—	1	6.9(4.6)	0	—	0	—	**2**	8.8(11.7)
Spinal fractures (excl pathological)	**201**	**2.3(143.9)**	68	1.5(12.9)	**93**	**2.1(51.0)**	**27**	**2.4(21.7)**	**174**	**2.4(135.3)**
Spinal fracture	**114**	**2.2(72.3)**	44	1.7(12.1)	**56**	**2.1(32.0)**	**21**	**3.1(30.3)**	**116**	**2.7(118.5)**
Cervical vertebral fracture	18	1.8(6.2)	7	1.4(0.8)	8	1.6(1.6)	**4**	**3.1(5.7)**	**17**	**2.0(8.7)**
Thoracic vertebral fracture	**21**	**2.5(17.9)**	**9**	**2.1(5.1)**	**15**	**3.5(25.7)**	1	0.9(<0.1)	**17**	**2.4(13.3)**
Lumbar vertebral fracture	**33**	**2.6(31.3)**	4	0.6(1.0)	**17**	**2.6(16.5)**	1	0.6(0.3)	17	1.6(3.5)
Fractured sacrum	**17**	**3.7(30.3)**	**6**	**2.5(5.2)**	**8**	**3.3(12.2)**	1	1.6(0.2)	**17**	**4.4(41.1)**
Fractured coccyx	6	1.6(1.4)	3	1.6(0.7)	2	1.1(<0.1)	1	2.1(0.6)	4	1.3(0.3)
Pelvic fractures	**88**	**2.5(76.8)**	20	1.1(0.2)	40	2.2(26.1)	**20**	**4.4(52.0)**	59	1.9(28.2)
Pelvic fracture	**65**	**2.2(38.5)**	18	1.2(0.5)	30	1.9(13.2)	**17**	**4.4(43.6)**	**51**	**2.0(24.9)**
Ilium fracture	3	3.0(3.6)	0	—	1	1.9(0.4)	1	7.5(5.6)	1	1.1(<0.1)
Pubis fracture	**12**	**4.5(55.7)**	2	1.4(0.2)	**6**	**4.3(14.3)**	2	5.5(7.3)	5	2.1(2.9)
Acetabulum fracture	**9**	**6.4(35.3)**	0	—	**5**	**6.6(22.0)**	1	5.0(3.1)	3	2.3(2.1)

	Omeprazole		Lansoprazole		Pantoprazole		Rabeprazole		Esomeprazole	
	Reports	PRR(χ^2)	Reports	PRR(χ^2)	Reports	PRR(χ^2)	Reports	PRR(χ^2)	Reports	PRR(χ^2)
Fractured ischium	1	1.4(0.1)	0	—	0	—	0	—	1	1.7(0.3)
Upper limb fractures	295	1.9(126.3)	109	1.4(12.8)	**157**	**2.0(77.0)**	37	1.9(14.8)	255	1.9(120.5)
Upper Limb fracture	102	1.7(27.5)	30	1.0(<0.1)	45	1.5(6.2)	9	1.2(0.2)	**118**	**2.4(88.1)**
Clavicle fracture	22	1.8(8.0)	7	1.2(0.1)	**17**	**2.8(19.0)**	3	1.9(1.3)	16	1.6(3.3)
Scapula fracture	**5**	**2.6(4.5)**	**5**	**5.2(16.0)**	2	2.0(0.9)	2	7.9(11.8)	2	1.2(0.1)
Humerus fracture	**46**	**2.4(34.9)**	18	1.8(6.7)	**35**	**3.6(61.9)**	**7**	**2.8(7.9)**	26	1.6(5.3)
Forearm fracture	**5**	**2.6(4.5)**	0	—	**5**	**5.1(15.5)**	0	—	2	1.2(0.1)
Radius fracture	**28**	**3.0(34.1)**	**10**	**2.1(5.4)**	**17**	**3.5(29.0)**	**7**	**5.7(26.4)**	11	1.3(0.9)
Ulna fracture	**11**	**2.9(12.5)**	**7**	**3.6(12.8)**	**6**	**3.0(7.8)**	0	—	**9**	**2.8(9.7)**
Wrist fracture	**82**	**2.1(45.8)**	33	1.7(8.9)	33	1.6(8.2)	**11**	**2.2(7.0)**	**69**	**2.1(39.0)**
Hand fracture	**39**	**2.1(20.4)**	15	1.6(3.1)	19	1.9(8.7)	3	1.1(0.1)	**37**	**2.3(27.3)**
Scapulothoracic dissociation	0	—	0	—	0	—	0	—	0	—
Lower limb fractures	633	1.6(124.2)	287	1.4(34.4)	338	1.6(81.9)	78	1.5(12.8)	558	1.6(137.3)
Lower limb fracture	67	1.3(5.3)	25	1.0(<0.1)	38	1.5(5.8)	8	1.2(0.4)	80	1.9(33.7)
Hip fracture	181	1.7(55.4)	83	1.6(18.1)	**116**	**2.2(73.8)**	**27**	**2.0(13.8)**	147	1.7(39.9)
Femoral neck fracture	29	1.5(4.8)	**23**	**2.4(18.6)**	18	1.8(6.8)	1	0.4(0.9)	20	1.2(0.9)
Femur fracture	205	1.6(49.2)	59	0.9(0.3)	86	1.3(7.3)	17	1.1(<0.1)	138	1.3(9.7)
Patella fracture	13	1.7(3.7)	5	1.3(0.4)	4	1.0(<0.1)	**5**	**5.2(16.5)**	11	1.7(3.2)
Tibia fracture	**40**	**2.4(29.5)**	**25**	**2.9(30.9)**	15	1.7(4.2)	3	1.3(0.3)	18	1.2(0.8)
Fibula fracture	**28**	**2.4(20.7)**	**13**	**2.2(7.9)**	**17**	**2.8(18.9)**	**6**	**3.9(12.7)**	16	1.6(3.3)
Ankle fracture	80	1.5(12.1)	47	1.7(14.8)	34	1.2(1.4)	10	1.4(1.4)	84	1.9(32.8)
Foot fracture	89	1.5(15.9)	48	1.7(12.3)	**62**	**2.1(35.1)**	**18**	**2.4(14.9)**	**118**	**2.4(88.1)**
Fractures NEC (excl pathological)	**239**	**2.1(122.8)**	**142**	**2.4(116.8)**	**139**	**2.3(102.6)**	**38**	**2.5(34.4)**	**207**	**2.1(117.1)**
Periprosthetic fracture	2	6.2(7.6)	0	—	2	12.4(18.2)	0	—	0	—

	Omeprazole		Lansoprazole		Pantoprazole		Rabeprazole		Esomeprazole	
	Reports	PRR(χ^2)	Reports	PRR(χ^2)	Reports	PRR(χ^2)	Reports	PRR(χ^2)	Reports	PRR(χ^2)
Fracture displacement	**10**	**6.1(37.2)**	**3**	**3.4(4.8)**	**4**	**4.5(10.2)**	2	8.8(13.4)	**7**	**4.9(19.9)**
Compression fracture	**65**	**3.6(112.6)**	**35**	**3.8(69.2)**	**48**	**5.2(152.7)**	**13**	**5.4(46.0)**	**53**	**3.5(87.1)**
Bone fragmentation	**12**	**4.0(24.7)**	**15**	**10.5(114.1)**	**8**	**5.2(25.2)**	1	2.5(0.9)	**10**	**3.9(20.3)**
Avulsion fracture	**4**	**6.2(15.2)**	1	2.8(1.2)	1	2.8(1.1)	0	—	1	1.7(0.3)
Jaw fracture	**25**	**2.2(15.5)**	**28**	**5.0(85.7)**	**23**	**4.0(49.5)**	2	1.3(0.2)	**21**	**2.2(13.1)**
Bone fissure	2	1.2(0.1)	0	—	1	1.2(<0.1)	2	9.5(14.9)	1	0.7(0.1)
Complicated fracture	**3**	**9.3(18.1)**	1	5.5(3.4)	1	5.4(3.3)	0	—	1	3.2(1.4)
Epiphyseal fracture	0	—	0	—	0	—	0	—	1	12.1(8.2)
Fracture	58	1.3(4.1)	28	1.3(1.6)	29	1.3(1.8)	8	1.4(1.0)	60	1.6(14.0)
Impacted fracture	1	8.1(5.2)	0	—	0	—	0	—	0	—
Multiple fractures	20	1.6(4.2)	**16**	**2.6(14.9)**	9	1.4(1.0)	2	1.2(0.1)	**21**	**2.0(10.2)**
Open fracture	2	0.8(0.1)	1	0.8(<0.1)	**5**	**4.1(11.0)**	0	—	**6**	**3.0(7.4)**
Stress fracture	**46**	**2.0(23.3)**	**26**	**2.3(18.6)**	21	1.8(7.4)	**10**	**3.4(17.0)**	**41**	**2.2(24.8)**
Torus fracture	0	—	1	20.6(14.9)	0	—	0	—	0	—
Traumatic fracture	3	1.2(0.1)	**5**	**4.0(10.9)**	1	0.8(0.1)	1	3.1(1.4)	1	0.5(0.6)
Greenstick fracture	0	—	0	—	0	—	0	—	0	—
Pathological fractures and complications	**122**	**3.1(157.6)**	**42**	**2.0(21.9)**	**62**	**3.0(79.2)**	**15**	**2.8(17.7)**	**81**	**2.4(61.7)**
Pathological fracture	**86**	**3.8(161.1)**	**29**	**2.4(24.1)**	**48**	**4.0(104.7)**	**7**	**2.3(5.0)**	**54**	**2.8(57.1)**
Pseudarthrosis	**4**	**3.2(5.7)**	**3**	**4.8(8.7)**	1	1.5(0.2)	0	—	0	—
Osteoporotic fracture	**11**	**2.2(6.9)**	2	0.8(0.1)	5	1.9(2.2)	2	3.1(2.8)	**12**	**2.9(14.2)**
Fracture nonunion	**15**	**2.3(10.4)**	**7**	**2.1(4.0)**	7	2.1(3.8)	**4**	**4.7(11.6)**	**14**	**2.6(12.7)**
Fracture malunion	0	—	0	—	1	2.4(0.8)	0	—	1	1.5(0.1)
Fracture delayed union	7	1.6(1.7)	1	0.5(0.6)	2	0.9(<0.1)	**5**	**9.3(36.1)**	**9**	**2.6(8.2)**
Synostosis	0	—	1	2.8(1.2)	0	—	0	—	0	—

* Dexlansoprazole is not in table due to insufficient reports.
